# Case report: Expanding the phenotype of *ARHGEF17* mutations from increased intracranial aneurysm risk to a neurodevelopmental disease

**DOI:** 10.3389/fneur.2022.1017654

**Published:** 2022-10-20

**Authors:** Ethiraj Ravindran, Noor Ullah, Shyamala Mani, Elaine Guo Yan Chew, Moses Tandiono, Jia Nee Foo, Chiea Chuen Khor, Angela M. Kaindl, Saima Siddiqi

**Affiliations:** ^1^Charité–Universitätsmedizin Berlin, Institute of Cell Biology and Neurobiology, Berlin, Germany; ^2^Charité–Universitätsmedizin Berlin, Department of Pediatric Neurology, Berlin, Germany; ^3^Charité–Universitätsmedizin Berlin, Center for Chronically Sick Children (Sozialpädiatrisches Zentrum, SPZ), Berlin, Germany; ^4^Institute of Biomedical and Genetic Engineering (IBGE), Islamabad, Pakistan; ^5^Khyber Medical University Institute of Paramedical Sciences (KMU IPMS), Peshawar, Pakistan; ^6^Human Genetics, Genome Institute of Singapore, A^*^STAR, Singapore, Singapore; ^7^Lee Kong Chian School of Medicine, Nanyang Technological University Singapore, Singapore, Singapore; ^8^Singapore Eye Research Institute, Singapore, Singapore

**Keywords:** ARHGEF17, neurodevelopmental disorder, microcephaly, motor dysfunction, missense mutation

## Abstract

RhoGTPase regulators play a key role in the development of the nervous system, and their dysfunction can result in brain malformation and associated disorders. Several guanine nucleotide exchange factors (GEF) have been linked to neurodevelopmental disorders. In line with this, ARHGEF17 has been recently linked as a risk gene to intracranial aneurysms. Here we report siblings of a consanguineous Pakistani family with biallelic variants in the *ARHGEF17* gene associated with a neurodevelopmental disorder with intellectual disability, speech delay and motor dysfunction but not aneurysms. Cranial MRI performed in one patient revealed generalized brain atrophy with an enlarged ventricular system, thin corpus callosum and microcephaly. Whole exome sequencing followed by Sanger sequencing in two of the affected individuals revealed a homozygous missense variant (g.11:73021307, c.1624C>T (NM_014786.4), p.R542W) in the *ARHGEF17* gene. This variant is in a highly conserved DCLK1 phosphorylation consensus site (I/L/V/F/M]RRXX[pS/pT][I/L/M/V/F) of the protein. Our report expands the phenotypic spectrum of *ARHGEF17* variants from increased intracranial aneurysm risk to neurodevelopmental disease and thereby add *ARHGEF17* to the list of GEF genes involved in neurodevelopmental disorders.

## Introduction

Neurodevelopmental disorders encompass a broad range of symptoms such as developmental delay, intellectual disability (ID), motor disorders, attention deficit and autism spectrum disorders (1). Such disorders can be caused by a disruption of the array of spatially and temporally regulated gene products that orchestrate nervous system development (2, 3). An important group of proteins known to contribute to pre- and postnatal brain development are the regulators of Rho family of GTPases (4).

Guanine nucleotide exchange factors (GEFs) stimulate the exchange of GDP for GTP and when bound to GTP the small GTPases bind various effectors to influence processes such as cell cycle progression, cell survival, cytoskeleton organization, and vesicular and nuclear transport (5). Several GEFs have been reported to regulate these processes during brain development, and biallelic variants in Rho guanine nucleotide exchange factor (*ARHGEF*) genes have been associated with human neurodevelopmental disorders: midbrain-hindbrain malformation (*ARHGEF2*) (6), nonsyndromic intellectual disability (*ARHGEF6*) (7), epileptic encephalopathy (*ARHGEF9*) (8), peripheral demyelinating neuropathy (*ARHGEF10*) (9). Recently, *ARHGEF17* has been reported as a risk gene for intracranial aneurysms (IA) (10). Here we report siblings of a consanguineous Pakistani family with biallelic variants in the *ARHGEF17* gene associated with a neurodevelopmental disorder with intellectual disability, speech delay and motor dysfunction but not aneurysms.

ARHGEF17 is a member of the RhoGEF (Rho GTPase GEF) of the diffuse B-cell lymphoma (Dbl) family (11). Members of this large protein family regulate the GDP-GTP cycling of specific proteins through a catalytic Dbl homology (DH) domain and have a regulatory pleckstrin homology (PH) domain that binds to phosphatidylinositol lipids (12). In addition, ARHGEF17 contains a WD40 domain that is important for protein-protein interactions and an actin binding domain (ABD) that binds to actin thereby controlling intracellular localization and its activity. Thus, distinct domains and sequences of ARHGEF17 exert autoregulation of its activity in a spatio-temporal manner and control cellular processes implicated during brain development (13, 14).

In this study, we report novel biallelic *ARHGEF17* variants and expand the phenotypic spectrum of *ARHGEF17* variants from increased intracranial aneurysm risk to neurodevelopmental disease.

## Subjects and methods

The human study was approved by the ethics committee of the Institute of Biomedical and genetic Engineering, Islamabad. The written informed consent was obtained for the molecular genetic analysis, the publication of clinical data, photos, and magnetic resonance images (MRI) from the index family. Blood samples were drawn from seven individuals from the affected family that included three affected (III.2, III.3, and III.4) and four unaffected (II.5, II.6, III.1 and III.5) individuals. Medical history but no clinical data were available from III.4 and III.6.

## Genotyping

Genomic DNA was extracted using standard methods and samples were genotyped on the Illumina OmniExpress 24v1-0-A BeadChip array. We confirmed the reported familial relationships among genotyped samples using PLINK identity by descent (IBD) analysis (–genome) (15, 16). We then scanned the data for homozygous segments (>1 Mb) which are shared among affected individuals but not for the unaffected individuals. Homozygosity mapping was conducted using PLINK v1.07 using the default settings (15).

## Whole exome sequencing

Targeted enrichment was performed on 1 μg of genomic DNA from two affected individuals (III.2 and III.3) using the Nimblegen SeqCap EZ Exome v3 kit and barcoded with four other samples for multiplexed 2 × 101 bp sequencing on a single lane of the Illumina HiSeq 2000 System. Each individual was sequenced to a mean coverage of 84.3–91.2 reads per target base, with 98% of the target exome covered by 10 or more reads. Reads were mapped using BWA v1.7. Variants were called using the GATK v2 Unified Genotyper following the recommended guidelines by GATK “Best practices for variant calling v3” (17). We used the following primer sequences for the confirmation of the identified *ARHGEF17* variant through Sanger sequencing: 5'-*AGGCACCTCTAGGGCATTG-3'* and 5'-*ACATCCCCTGCCCAGTC-3'*.

## Homozygosity mapping

Homozygous segments of >1 Mb in length accounted for 8.8–15.8% of the genome in all three affected individuals and their two non-affected siblings, confirming that these individuals are the offspring of a consanguineous union. We identified three large segments that were homozygous only in affected individuals (Chr4q11-q13.3 LOD 2.7, Chr11q13.4-q13.5 LOD 2.7 and Chr15q14-q21.2 LOD 2.7).

## Genetic analysis

For confirmation of causal mutation, we performed whole exome sequencing analysis in two affected siblings (III.2 and III.3) to identify a set of homozygous mutations that are shared between the two siblings within the homozygous segment. A total of 38,237 variants were found in these two individuals, out of which 26,042 were coding and 13,145 were nonsynonymous, frameshift or splice site variants in well-annotated transcripts. Of these, 902 were rare, either absent or present in < 1% of all populations in HapMap, 1000 genomes populations (17) and the NHLBI exome variant server (EVS) databases (URL: http://evs.gs.washington.edu/EVS/). Of these, 12 variants were homozygous in both affected siblings and six resided within the shared homozygous intervals on chromosomes 4, 11 and 15. Only two were predicted to be damaging: one in *ARHGEF17* [Chr11:73021307, hg19/GRCh37, c.1624 C>T (NM_014786.4)] and one in Kinase insert domain receptor (*KDR*) [Chr4:55955592, hg19/GRCh37, c.3352 C>T (NM_002253.4)]. While both mutations were present in gnomAD South Asians, the *KDR* variant (allele frequency of 0.24%) is present at higher frequencies than the *ARHGEF17* variant (allele frequency of 0.0098%). KDR has been shown to play an essential role in the regulation of angiogenesis, vascular development, vascular permeability, and embryonic hematopoiesis and heterozygous variants in *KDR* has been linked to Hemangioma (MIM# 602089). Since members of the ARHGEF family have been associated to neurodevelopmental disorders, we consider ARHGEF17 as a strong candidate for the phenotype observed in our index patients.

## Results

### Clinical presentation

We report four individuals of a consanguineous family of Pakistani descent with a neurodevelopmental disorder. Two affected individuals (III.2, III.3) were available for assessment. Two further individuals were reported to have been affected by a similar developmental disease but were deceased due to a tetanus infection (III.4) and severe head injury (III.6) at ages 20 and 7 years, respectively, and were not available for evaluation.

**Figure 1:**
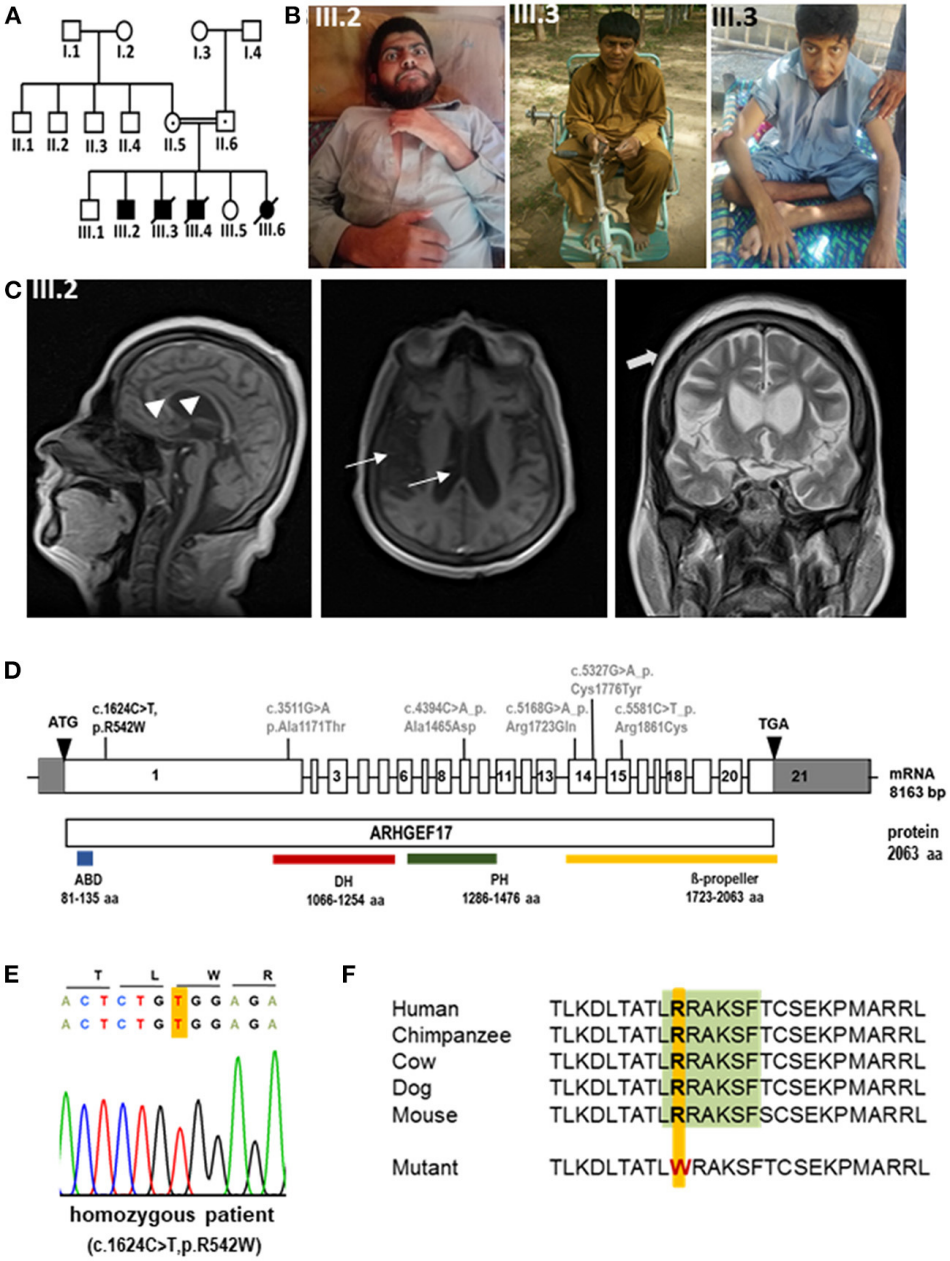
Phenotype and genotype of index patients with *ARHGEF17* mutation **(A)** Pedigree of index family indicating affected patients (III.2, III.3). **(B)** Photomicrographs of III.2 and III.3. **(C)** MRI images of III.2 revealed general atrophy/hypoplasia of the brain parenchyma, enlarged ventricular system (thin arrows) and thin, but complete corpus callosum (arrow heads), chronic ischemic changes periventricular white matter surrounding occipital horns, moderate cerebral atrophy and thickened scull bone (thick arrows). **(D)** Schematic representation of *ARHGEF17* cDNA with 21 exons and the wild-type ARHGEF17 protein with the identified variants of our index family (black) and previously reported homozygous variants (gray). A homozygous exchange of a single base C to T in exon 1 of the *ARHGEF17* (c.1624C>T (NM_014786.4), p.R542W (NP_055601.2) was identified and confirmed through Sanger sequencing. **(E)** Electropherogram traces depicting the exchange of C to T at position 1624 of the *ARHGEF17* gene. **(F)** The site of mutation is located in DCLK1 consensus site [LRRAKSF (green)] of ARHGEF17 and it is highly conserved across species.

Proband 1 (P1, III.2) was a 24-year-old male born at term without complications with normal body weight and length (Figures 1A,B, Table 1). He had developmental delay, and later moderate-to-severe intellectual disability and a speech disorder was diagnosed. He began to speak at 3.5 years of age but could not communicate properly. He could understand only simple commands. He was able to walk at the age of 1 year. No dysmorphism or cranial nerve paralysis were noted on clinical examination. He had hypotonia. No head circumference data was available at birth. At the age of 12 years, the proband experienced frequent falls and gradually lost the ability to walk. He could not lift his extremities and became restricted to his bed. He was able to swallow food but could not eat or drink without any support. No gastrostomy was performed. P1 had progressive loss of muscle strength with muscle weakness and wasting, but no joint contractures. Tendon reflexes were brisk, no pathological reflexes were present. There were no coordination problems, and no tremor. Pes equinus was noted. He lost the ability to communicate at the age of 12 years. No visual or hearing impairments were noted. Echocardiogram did not show any abnormality, particularly no sign of cardiomyopathy. Microcephaly was observed with an occipitofrontal head circumference (OFC) of 53.3 cm [ < 3^rd^ centile, < −2 standard deviations (SD)] at the age of 22 years. Postnatal OFC values were not available. MRI findings revealed generalized brain atrophy, accentuated at the temporal lobes, with incomplete opercularization, enlarged ventricular system and thinned corpus callosum (consequence of the parenchymal atrophy), chronic ischemic changes periventricular white matter surrounding occipital horns, and thickened skull bone (Figure 1C).

**Table 1:** Clinical phenotype of index patients with biallelic *ARHGEF17* variant c.1624C>T (NM_014786.4).

	**III.2**	**III.3**
Gender	Male	Male
Age at diagnosis (years)	12	12
Age at last diagnosis (years)	24	22
Age at death (years)	Alive	24
Pregnancy	No complications	No complications
Delivery	No complications	No complications
Birth at gestational age (weeks)	38	38
Birth weight	Normal	Normal
Birth length	Normal	Normal
Head circumference	(-2 SD, 53.3 cm)	(-0.5 SD, 55.9 cm)
Microcephaly	Yes, primary	No
Developmental delay	Yes	Yes
Speech delay	Yes	Yes
Intellectual disability	Moderate-severe (understands simple commands)	Moderate-severe
Visual impairment	No	No
Gross Motor Functional Classification Scale (GMFCS)	GMFCS level 5	GMFCS level 5
Muscle strength	Reduced paraplegia of lower limbs (progressive falling at 12 y/o)	Reduced paraplegia of lower limbs (progressive falling at 12 y/o)
Muscle trophic	Atrophic	Atrophic
Orthopedic	Pes equinus	-
Echocardiography	Normal	Normal

Proband 2 (P2, III.3) was a 22-year-old male born at term with normal birth weight and length (Figures 1A,B, Table 1). He had developmental delay, and later moderate-to-severe intellectual disability and a speech disorder were diagnosed. He began to speak at 3.5 years of age but could not communicate properly. He was able to walk by 1 year of age. Clinical examination showed no dysmorphism or cranial nerve paralysis. Similar to his brother, frequent falls started at the age of 12 years, he developed progressive loss of muscle strength with muscle weakness and wasting. He could not lift his extremities and lost the ability to walk, and became wheelchair bound. He was able to swallow food but could not eat or drink without any support. No gastrostomy was performed. He became wheelchair bound. He could not communicate at the age of diagnosis. No visual or hearing impairments were noted. Echocardiogram did not show any abnormality, particularly no signs of cardiomyopathy. No microcephaly was observed (55.9 cm, −0.5 SD). No MRI was performed. He died at the age of 24 years due to unknown cause in an episode of a febrile illness and abdominal pain.

### Genetic findings

Through whole exome sequencing, we identified the homozygous exchange of a single base C to T in exon 1 of the *ARHGEF17* gene (Chr 11:73021307, hg19/GRCh37, c.1624C>T, NM_014786.4; p.R542W, NP_055601.2) in two affected siblings (III.2 and III.3) (Figure 1D) and confirmed the mutation by Sanger sequencing (Figure 1E). The identified variant lies in a protein region highly conserved across species (Figure 1F). The variant is disease-causative in nature, as predicted by Mutation Taster and SIFT (https://www.mutationtaster.org/, http://genetics.bwh.harvard.edu/pph2/, https://sift.bii.a-star.edu.sg).

## Discussion

Here we describe the novel biallelic variant c.1624C>T in the *ARHGEF17* gene (NM_014786.4) to be associated with a neurodevelopmental disorder with intellectual disability, speech disorder and progressive motor dysfunction. *ARHGEF17* is highly expressed in the brain throughout development but not in skeletal muscle according to the EMBL-EBI expression atlas (https://www.ebi.ac.uk/gxa/home; ensg00000110237) (11). Given this expression pattern of *ARHGEF17*, muscle weakness and wasting in light of brisk reflexes observed in index patients may result from a central nervous system effect rather than a neuropathy or myopathy. Unfortunately, no electrophysiology or muscle biopsy data was available to further discriminate the cause of progressive motor dysfunction.

A previous report had identified five variants in the *ARHGEF17* gene in individuals with intracranial aneurysms (IA) and classified the gene as a genetic risk factor for IA (10). All the reported variants affected the C-terminus and mapped to the known functional DH, PH and WD40 domains (Figure 1D). Morpholino-based knockdown of *Arhgef17* in zebrafish (*Danio rerio*) led to intracranial hemorrhage and erythrocyte extravasation, further supporting the conclusion that *ARHGEF17* mutations are a risk factor for IA (10). The current identified variant of the index patients resides in the N-terminus and results in an amino acid change of arginine to tryptophan at position 542. This results in the disruption of a DCLK1 (doublecortin-like kinase 1) phosphorylation consensus motif I/L/V/F/M]RRXX[pS/pT][I/L/M/V/F. Sequence alignment showed that the DCLK1 consensus site in ARHGEF17, LRRAKSF, is highly conserved across species, including *H. sapiens, P. troglodytes, M. mulatta, C. lupus, B. taurus, M. musculus, R. norvegicus*, and *G. gallus*. Intriguingly, the N-terminus of *ARHGEF17* is present neither in *Danio rerio* nor *Xenopus tropicalis*, suggesting that the additional functions were acquired by the N-terminus during evolution. DCLK1 is a protein very similar to doublecortin (DCX) that has been implicated in neural development including cortical malformation in humans (lissencephaly, subcortical laminal heterotopia) (18, 19). DCLK1 plays an important role in controlling mitosis and spindle organization as well as in neuronal cell fate (19) and suggests a mechanism by which the novel *ARHGEF17* variant described here could result in a neurodevelopmental disorder. In line with this, a heterozygous variant in the *DCLK1* gene has been reported in an individual with abnormalities in the musculoskeletal and nervous system in the DECIPHER database (https://www.deciphergenomics.org/gene/DCLK1/ddd-research-variant-overlap).

Considering the severity of the phenotype in the index patients during development and with age and the indirect cause of death of three patients might suggests that ARHGEF17 is implicated in neurodevelopmental as well as in neurodegenerative disorder. ARHGEF17 has been linked to intracranial aneurysm which is in line with its higher expression levels in blood vessels and maintenance of the integrity of blood vessels (11). In support of this, studies have shown the importance of vascular functions during early stages of brain development and cognitive functioning throughout adulthood (20–22). Vascular dysfunctions have been associated to various neurodevelopmental (autism spectrum disorders, microcephaly, schizophrenia) and neurodegenerative disorders (Alzheimer's disease, Parkinson's disease, multiple sclerosis) (20–22).

In conclusion, we have identified a new variant in *ARHGEF17* in individuals with a neurodevelopmental disorder. Identification of additional families and functional experiments are necessary to delineate the role of ARHGEF17 in brain and musculoskeletal development. Our report expands the phenotypic spectrum of ARHGEF17-associated human disorders and adds *ARHGEF17* to the list of GEF genes linked to neurodevelopmental diseases.

## Data availability statement

The datasets presented in this article are not readily available because of ethical and privacy restrictions. Requests to access the datasets should be directed to the corresponding authors.

## Ethics statement

The studies involving human participants were reviewed and approved by Ethics Committee of the Institute of Biomedical and Genetic Engineering, Islamabad. Written informed consent to participate in this study was provided by the participants' legal guardian/next of kin. Written informed consent was obtained from the individual(s), and minor(s)' legal guardian/next of kin, for the publication of any potentially identifiable images or data included in this article.

## Author contributions

SS was responsible for project conception. AK analyzed and interpreted patient's clinical and genetic data. NU and SS contributed clinical samples by recruiting subjects, gathering patient history, clinical information, and written informed consents. EC, MT, JF, and CK performed WES and bioinformatics data analysis. EC, MT, JF, and SS performed Sanger sequencing and segregation analysis. SM interpreted the genetic data and performed bioinformatic analysis. SM, ER, and AK drafted the manuscript that was revised and accepted by all coauthors. All authors contributed to the article and approved the submitted version.

## Funding

We acknowledge the funding resources for this study: The genetic analysis was supported by the Agency for Science, Technology and Research, Singapore (to CK) and a Singapore National Research Foundation Fellowship (NRF-NRFF2016-03; to JF). Family and clinical data collection was supported by departmental grant (IBGE) (SS). Further funding includes the German Research Foundation (SFB665, SFB1315, FOR3004, AK), the Sonnenfeld Stiftung (AK), the Berlin Institute of Health (BIH, CRG1, AK) and the Charité (AK, ER, and SM). This study makes use of data generated by the DECIPHER community available on the DECIPHER webpage (www.deciphergenomics.org).

## Conflict of interest

Author CK was employed by Singapore Eye Research Institute. The remaining authors declare that the research was conducted in the absence of any commercial or financial relationships that could be construed as a potential conflict of interest.

## Publisher's note

All claims expressed in this article are solely those of the authors and do not necessarily represent those of their affiliated organizations, or those of the publisher, the editors and the reviewers. Any product that may be evaluated in this article, or claim that may be made by its manufacturer, is not guaranteed or endorsed by the publisher.
